# Supplementary material for: Sustained-input switches for transcription factors and microRNAs are central building blocks of eukaryotic gene circuits
Source: Genome Biol. 2013 Aug 23;14(8):R85. doi: 10.1186/gb-2013-14-8-r85 (PMC4054853; doi:10.1186/gb-2013-14-8-r85)
Supplement: Additional file 5 — HTML Browsable Motif Output. Zipped folder containing all WaRSwap and FANMOD motif output, viewable in a web browser. [file gb-2013-14-8-r85-S5.ZIP › HTML_browsable_motif_output/WaRSwap_ath_tair10_withSelfLoops/sigs_WSWRSLDraw_TAIR10_SLColors-2500.pvals.heatmaps.html/motif_id_46_100131113_tftype_ath_upstream_-3000_0.html]

```
BG_MODEL = WARSwap
MOTIF_ID = 46_100131113
TF_TYPE = ath
UPSTREAM = -3000_0


PVals
FNR = 0.2	FNR = 0.4	FNR = 0.6	FNR = 0.8
deltaG = 60	0.01	0.015	0	0.162
deltaG = 70	0.013	0.012	0	0.144
deltaG = 80	0.01	0.015	0	0.145

ZScores
FNR = 0.2	FNR = 0.4	FNR = 0.6	FNR = 0.8
deltaG = 60	1.956	2.311	5.132	-0.369
deltaG = 70	1.961	2.415	4.99	-0.327
deltaG = 80	1.952	2.29	5.199	-0.337

StDevs
FNR = 0.2	FNR = 0.4	FNR = 0.6	FNR = 0.8
deltaG = 60	2462.141	1114.913	135.769	7.045
deltaG = 70	2497.56	1067.48	139.237	7.573
deltaG = 80	2523.141	1122.204	135.274	7.318
```
